# Thermoelectric transport in temperature-driven two-dimensional topological insulators

**DOI:** 10.1038/s41598-017-08084-w

**Published:** 2017-08-08

**Authors:** H. M. Dong, L. L. Li, W. Xu, J. L. Liu

**Affiliations:** 1 School of Physics, China University of Mining and Technology, Xuzhou, 221116 P.R. China; 2 Key Laboratory of Materials Physics, Institute of Solid State Physics, Chinese Academy of Sciences, Hefei, 230031 China; 3 Department of Physics, Yunnan University, Kunming, 650091 P.R. China; 4 School of Materials Science and Engineering, China University of Mining and Technology, Xuzhou, 221116 P.R. China

## Abstract

We theoretically investigate on the thermoelectric (TE) transport properties of edge and bulk states in a temperature-driven two-dimensional (2D) topological insulator (TI) realized from CdTe/HgTe/CdTe quantum wells (QWs). It is found that the temperature can effectively drive a TI phase in CdTe/HgTe/CdTe QWs. We find that the TE transport properties of 2D TI can be governed by edge states, bulk states, or their interplay, depending on driving temperature and chemical potential of the system. Moreover, we find that the TE figure of merit *ZT* shows a peak at relatively low temperatures due to the competition between bulk and edge transports. This peak vanishes at relatively high temperatures due to the dominance of bulk states in the TE transport. With decreasing the ribbon width of the temperature-driven 2D TI, the low-temperature *ZT* exhibits two peaks, among which one occurs due to the bulk-edge competition and the other occurs due to the edge-edge hybridization; while the high-temperature *ZT* first exhibits the bulk-state behavior and then the edge-state one, which is indicative of a bulk-to-edge transition in the TE transport.

## Introduction

In recent years, two-dimensional (2D) and three-dimensional (3D) topological insulators (TIs) have drawn a great attention in both condensed matter physics and material science^1, 2^. TIs are a new class of quantum materials with strong spin-orbit couplings (SOC), which leads to the formation of topological edge (in 2D case) and surface (in 3D case) states. These boundary (edge or surface) states protected by time-reversal (TR) symmetry are topologically robust against TR-invariant perturbations, and their spin orientations are locked to their momentum directions due to spin-momentum lockage^3^. Since CdTe/HgTe quantum wells (QWs) were first discovered as 2D TIs^4, 5^, many other 2D and 3D TIs have been theoretically predicted and experimentally confirmed in different semiconductor systems^6–10^. Rich and interesting physics, such as quantum spin Hall effect^4, 5^, finite-size effect^11, 12^, weak localization/anti-localization^13, 14^, magneto-electric/magneto-optical effects^15, 16^, electron correlation^17, 18^, elementary excitation^19, 20^, nonlinear optical response^21, 22^, etc, in 2D and 3D TIs have been investigated. At present, research work in looking for new TI materials and exploring their interesting physical properties is still active.

Recently, 2D and 3D TIs have been proposed as high-performance thermoelectric (TE) materials^23–26^. The physical reason is that by introducing strong disorder into the TIs, the electron conduction carried by edge or surface states remains good since these boundary states are topologically robust against disorder while the phonon conduction could be largely suppressed due to enhanced phonon scattering by disorder. Thus, one may expect that the TE figure of merit *ZT* can be greatly improved. However, the surface states of 3D TIs are not topologically protected against disorder-induced scattering at any angle other than backscattering, although the topological robustness of edge states has been verified in 2D TIs based on HgTe QWs^27^. Therefore, from this perspective, 2D TIs are more preferable for the design of high-performance TE materials. Furthermore, 2D TIs are also low-dimensional systems, which is also beneficial for the design^28^: the use of quantum confinement to enhance the Seebeck coefficient and the use of numerous interfaces to enhance phonon scattering.

In the most of existing research works, although the authors have investigated the TE transport properties of both edge and bulk states in 2D TIs, they have commonly used a rough and imprecise band structure model (e.g., the linear and parabolic energy dispersions for the edge and bulk states, respectively) with some free parameters such as bulk-electron effective mass, bulk-state band gap, and edge-state hybridization gap. As is well known, in 2D TIs realized from CdTe/HgTe QWs, these important parameters depend strongly on quantum structure parameters such as QW thickness^4^ and ribbon width^11^. In our previous work, we started from a realistic band structure model and studied the influence of QW thickness and ribbon width on the TE transport properties of 2D TIs based on CdTe/HgTe QWs^29^. Unfortunately, in that work we only focused on the TE transport properties of 2D TIs at a relatively low temperature (*T* = 60 K). It is known that the temperature could be very important for the practical application of TE devices. In the present work, we extend the previous band structure model^29^ to include the temperature effect and study the influence of temperature on the TE transport properties of 2D TIs based on CdTe/HgTe QWs. The band structure calculation shows that the temperature can effectively drive a topological phase transition in CdTe/HgTe QWs. As a result, such important parameters as bulk-electron effective mass, bulk-state band gap, and edge-electron group velocity are strongly dependent on temperature. Based on these results, one may expect that the temperature should have a substantial influence on the TE transport properties of 2D TIs based on CdTe/HgTe QWs. This is the prime motivation of the present work. In this work, we present a systematic theoretical study of the thermoelectric properties of 2D TIs. We develop a simple and transparent theoretical approach to investigate the effects of temperature, chemical potential, and ribbon width on the thermoelectric transport properties of edge and bulk states in a 2D TI.

The paper is organized as follows. In Section II, we outline the theoretical approach to calculate the band structures and TE transport properties of temperature-driven 2DTIs realized from CdTe/HgTe QWs. In Section III, the numerical results are presented and discussed. Finally, the concluding remarks are given in Section IV.

## Theoretical Approach

### Band structure calculation with temperature effect

We consider a symmetric CdTe/HgTe QW of thickness *d* grown along the [001] direction, i.e., the *z*-direction. The 2D-plane or (*x*, *y*)-plane of this QW is designed into a ribbon of width *w* along the *y*-direction and of length *l* along the *x*-direction. The schematic plot of such a model system can be found in our previous work^29^ and we do not present it here.

With the envelope function theory, the six-band Kane model is used to calculate the energy spectra and wave functions of CdTe/HgTe QWs. The model is established on the basis set consisting of six Bloch atomic orbitals: two *s*-like orbitals multiplied by spin and four *p*-like orbitals coupled with spin to form four orbitals with total angular momentum *J* = 3/2. There are totally seven input parameters used in the six-band Kane model^29^, i.e., *E*_*g*_ (band gap), *E*_*p*_ (parameter related to the Kane’s momentum matrix element), Δ_*so*_ (spin-orbit splitting energy), *A*_*c*_ (parameter related to the electron effective mass), and *γ*_*i*_ (*i* = 1, 2, 3, three Luttinger paramters). A number of empirical expressions for the temperature dependence of Hg_1−*x*_Cd_*x*_Te band gap are available at present^30–33^. The most widely used expression according to Hansen *et al*.^31^ is given by

1 $${E}_{g}=-0.302+1.930x-0.810{x}^{2}+0.832{x}^{3}+\mathrm{0.535(1}-2x)T\mathrm{/1000,}$$

where *E*_*g*_ is in units of eV and *T* is in units of K. This expression is valid for all alloy composition 0 ≤ *x* ≤ 1 and for temperatures 5 ≤ *T* ≤ 300 K^31^. The dependence of *E*_*g*_ on temperature automatically leads to temperature-dependent *A*_*c*_ via^29^

2 $${A}_{c}=1+{E}_{p}\mathrm{/[3(}{E}_{g}+{{\rm{\Delta }}}_{so}\mathrm{)]}.$$

Except for *E*_*g*_ and *A*_*c*_, no other parameters such as *E*_*p*_, Δ_*so*_, and *γ*_*i*_ (*i* = 1, 2, 3) are dependent on temperature, which follows the previous work^34^. The details of the application of six-band Kane model to CdTe/HgTe QWs can be found in ref. 29. Our numerical results show that the temperature has a significant impact on the band structure properties of CdTe/HgTe QW, and as will be seen in the Section. III, it can effectively drive a topological phase transition in CdTe/HgTe QWs.

With the obtained energy spectra and wave functions for electrons and holes in the CdTe/HgTe QW, we can construct an effective 2D Hamiltonian via reduction procedure^29^. By choosing the electron and hole ground states (each state is spin degenerate) as a basis set, the matrix form of this Hamiltonian is given by^29^

3 $$H({\bf{k}})=[\begin{array}{cccc}{E}_{e}+B{k}^{2} & A{k}_{+} & 0 & 0\\ {A}^{\ast }{k}_{-} & {E}_{h}+C{k}^{2} & 0 & 0\\ 0 & 0 & {E}_{e}+B{k}^{2} & -A{k}_{-}\\ 0 & 0 & -{A}^{\ast }{k}_{+} & {E}_{h}+C{k}^{2}\end{array}],$$

where **k** = (*k*_*x*_, *k*_*y*_) is the 2D wavevector, *k*_±_ = *k*_*x*_ ± *ik*_*y*_, *E*_*e*_ and *E*_*h*_ are the lowest electron and the highest hole energy levels at the center of Brillouin zone, respectively, *B* and *C* are related to the electron and heavy-hole effective masses, and *A* is determined by the overlap of wave functions for the lowest electron state and the highest hole state at the zone center. These parameters depend sensitively on temperature *T* and thickness *d*. As can be seen, the effective 2D Hamiltonian consists of two decoupled blocks, which are related by the TR symmetry.

The effective 2D Hamiltonian given by Eq. (3) is employed to calculate the energy spectra and wave functions for edge and bulk states in the 2D TI based on the CdTe/HgTe QW. For the 2D TI in the ribbon geometry, we assume the periodic boundary condition in the *x*-direction and confining boundary condition in the *y*-direction. Thus, *k*_*x*_ is a good quantum number but *k*_*y*_ is replaced by −*id*/*dy*. The energy spectra and wave functions for edge and bulk states are obtained by solving the following Schrödinger equation

4 $$H({k}_{x},-i\frac{d}{dy}){{\rm{\Phi }}}_{{k}_{x}}(x,y)=E({k}_{x}){{\rm{\Phi }}}_{{k}_{x}}(x,y),$$

where *E*(*k*_*x*_) is the energy spectrum and ${{\rm{\Phi }}}_{{k}_{x}}(x,y)={e}^{i{k}_{x}x}{[{\varphi }_{1}(y),{\varphi }_{2}(y),{\varphi }_{3}(y),{\varphi }_{4}(y)]}^{T}$ is the four-component wave function with *ϕ*_*j*_(*y*) (*j* = 1~4) being the envelop functions along the *y*-direction. Considering the hard-wall potential confinement^35^ *, ϕ*_*j*_(*y*) (*j* = 1~4) satisfies the following boundary condition

5 $${\varphi }_{j}(y=\pm w\mathrm{/2)}=0.$$

With such a boundary condition, we use the finite difference method^36^ to numerically solve the Schrödinger equation given by Eq. (4).

### TE transport coefficients

We now consider the TE transport properties of edge and bulk states in the 2D TI ribbon. The transport direction is taken along the *x*-direction. TE transport coefficients such as electrical conductivity *σ*, Seebeck coefficient *S*, and electron thermal conductivity *κ* can be expressed in terms of the transport integrals *L*_*j*_ (*j* = 0, 1, 2) as follows^24^

6a $$\sigma ={e}^{2}{L}_{0},$$

6b $$S=-\frac{1}{eT}\frac{{L}_{1}}{{L}_{0}},$$

6c $$\kappa =\frac{1}{T}\frac{{L}_{0}{L}_{2}-{L}_{1}^{2}}{{L}_{0}},$$

where *e* is the elementary charge. By neglecting the phononic contribution to the TE transport, the TE figure of merit *ZT* can be written as^24^

7 $$ZT=\frac{{L}_{1}^{2}}{{L}_{0}{L}_{2}-{L}_{1}^{2}}.$$

In general, the thermoelectric figure of merit *ZT* is written as

8 $$ZT=\frac{T\sigma {S}^{2}}{({\kappa }_{e}+{\kappa }_{p})}.$$

with T being the temperature and *κ*_*e*_ (*κ*_*p*_) the electron (phonon) thermal conductivity. It is clear that the inclusion of *κ*_*p*_ leads to a decrease for *ZT*. Therefore, when designing high-performance thermoelectric materials, one of the most efficient methods is to suppress the phonon thermal conductivity as largely as possible, which can enhance *ZT* markedly. In 2D TIs, the phononic thermal conductivity can be suppressed (*κ*_*p*_ → 0) by doping nonmagnetic impurities in the systems in order to achieve the higher *ZT*. The physical reason is discussed in these two references, i.e., strong phonon-nonmagnetic impurity or disorder scatterings can dramatically reduce phononic thermal conductivity^37, 38^.

The edge states in the 2D TI are perfectly conducting and they can be viewed as 1D ballistic transport channels. To describe the edge-state transport, we use the Landauer-Büttiker formula. The transport coefficients for edge states ${L}_{j}^{e}\,(j=\mathrm{0,}\,\mathrm{1,}\,\mathrm{2)}$ are given by^24^

9 $${L}_{j}^{e}=\frac{\lambda l}{sh}{\int }_{-\infty }^{+\infty }dET(E)(E-\mu {)}^{j}(-\frac{\partial f}{\partial E}),$$

where *λ* = 2 accounts for the spin degeneracy, *l* is the length of 2D TI ribbon, *s* is the cross-section area of 2D TI ribbon, *h* is the Plank constant, *T*(*E*) is the energy-dependent transmission coefficient, *μ* is the chemical potential of 2D TI ribbon, and $f(E)=\mathrm{1/[}{e}^{(E-\mu )/{k}_{B}T}+\mathrm{1]}$ is the Fermi-Dirac function with *k*_*B*_ being the Boltzmann constant. Here we consider the following three important features for edge states in the 2D TI ribbon: (1) when the electron energy *E* lies inside the bulk band gap Δ_*b*_, the transmission probability *T*(*E*) = 1 which reflects the perfectly conducting nature of edge states; (2) when the finite-size effect is taken into account, the edge states at two boundaries of the ribbon can hybridize substantially to open a finite gap Δ_*e*_ in the edge-state energy spectrum; and (3) due to presence of such a hybridization gap, the transmission coefficient is given by^11^

10 $$T(E)=\frac{1}{{e}^{\beta [({{\rm{\Delta }}}_{e}\mathrm{/2)}-\mu ]}+1}-\frac{1}{{e}^{\beta [(-{{\rm{\Delta }}}_{e}\mathrm{/2)}-\mu ]}+1}+\mathrm{1,}$$

where *β* = 1/(*k*_*B*_ *T*). Based on these considerations, the transport integrals ${L}_{j}^{e}$ (*j* = 0, 1, 2) can be written as

11 $${L}_{j}^{e}=\frac{\lambda l}{sh}[{\int }_{-{{\rm{\Delta }}}_{b}\mathrm{/2}}^{-{{\rm{\Delta }}}_{e}\mathrm{/2}}dE{F}_{j}(E)+{\int }_{{{\rm{\Delta }}}_{e}\mathrm{/2}}^{{{\rm{\Delta }}}_{b}\mathrm{/2}}dE{F}_{j}(E)]$$

with *F*_*j*_(*E*) = *T*(*E*)(*E*−*μ*)^*j*^(−∂*f*/∂*E*). Here we have taken the Dirac point in the edge-state energy spectrum as the zero of energy. By making integral variable substitution in Eq. (9), the transport integrals ${L}_{j}^{e}$ (*j* = 0, 1, 2) can be obtained as

12 $${L}_{j}^{e}=\frac{\lambda l}{sh}{({k}_{B}T)}^{j}[{\int }_{-{x}_{b}-{x}_{0}}^{-{x}_{e}-{x}_{0}}dx{ {\mathcal F} }_{j}(x)+{\int }_{{x}_{e}-{x}_{0}}^{{x}_{b}-{x}_{0}}dx{ {\mathcal F} }_{j}(x)],$$

where *x*_*b*_ = *β*Δ_*b*_/2, *x*_*e*_ = *β*Δ_*e*_/2, *x*_0_ = *βμ*, and ${ {\mathcal F} }_{j}(x)={x}^{j}{e}^{x}/[{({e}^{x}+\mathrm{1)}}^{2}]$.

The bulk-state transport is diffusive and the Boltzmann formalism applies. By solving the Boltzmann transport equation with relaxation time approximation, the transport integrals for bulk states ${L}_{j}^{b}(\,j=\mathrm{0,\; 1,\; 2)}$ are given by ref. 29

13 $${L}_{j}^{b}=\frac{\lambda }{s}{\int }_{-\infty }^{+\infty }dE{\rm{\Pi }}(E)(E-\mu {)}^{j}(-\frac{\partial f}{\partial E}),$$

where Π(*E*) is the so-called transport distribution function^39^, and is given by

14 $${\rm{\Pi }}(E)=\sum _{{k}_{x}}{v}^{2}({k}_{x})\tau ({k}_{x})\delta [E-E({k}_{x})].$$

Here, *τ*(*k*_*x*_) is the relaxation time for bulk electrons which is assumed to be a constant *τ*, and *v*(*k*_*x*_) is the group velocity of bulk states which is given by

15 $$v({k}_{x})=\frac{1}{\hslash }\langle {\rm{\Psi }}({k}_{x})|\frac{\partial H({\bf{k}})}{\partial {k}_{x}}|{\rm{\Psi }}({k}_{x})\rangle .$$

Taking into account of both edge-state and bulk-state contributions, the total transport integrals are obtained as

16 $${L}_{j}={L}_{j}^{e}+{L}_{j}^{b}(j=\mathrm{0,}\,\mathrm{1,}\,\mathrm{2)}.$$

## Results and Discussion

**Figure 1:**
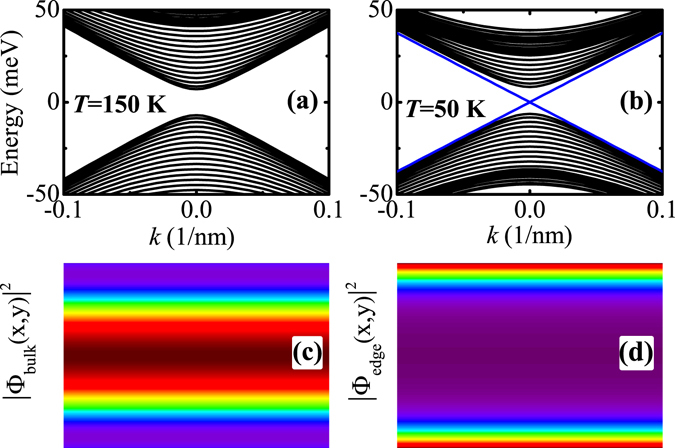
Demonstration of the TI phase transition driven by temperature: (**a**) and (**b**) plot the energy spectra of ribbon structures in NI and TI regimes, respectively, and (**c**) and (**d**) plot the probability density distributions for bulk and edge states at *k* = 0, respectively. Here, the blue solid lines and black solid curves in (**a**) and (**b**) represent the energy dispersions for edge and bulk states, respectively, and the purple and red colors in the rainbow maps in (**c**) and (**d**) represent the minimum and maximum values of probability density distribution, respectively.

In this paper, we calculate the band structures and TE transport properties of temperature-driven 2D TIs (in the ribbon geometry) realized from CdTe/HgTe QWs. For the band-structure calculation, the input parameters such as *E*_*g*_, *E*_*p*_, Δ_*so*_, *γ*_1_, *γ*_2_ and *γ*_3_ at *T* = 0 K are taken from ref. 40. These parameters are frequently used and well documented in the literature. The valence band offset between HgTe and CdTe is taken as 570 meV at *T* = 0 K^40^ and is assumed to be temperature independent. For the TE transport calculation, the input parameters such as *l*, *s*, and *τ* are required. We take the the length of 2D TI ribbon as *l* = 1 *μ*m^41^. The cross-section area of 2D TI ribbon is taken as *s* = 100 nm × 10 nm. The relaxation time for bulk electrons is taken as *τ* = 10^−14^ s, corresponding to a case with strong disorder. The temperature *T*, QW thickness *d* and ribbon width *w* are varied to investigate their influences on the electronic band structure and TE transport properties of 2D TI ribbon. The chemical potential *μ* considered in this work starts from zero. In Fig. 1, we show the demonstration of the TI phase transition driven by temperature. For a normal insulator case [see e.g. Fig. 1(a)], when the chemical potential *μ* is above (below) the band gap, it means a n-type (p-type) system in which the TE transport is governed by the electrons (holes). However, for a topological insulator case [see e.g. Fig. 1(b)], when the Fermi energy is located within the band gap, it indicates an edge-state-dominated TE transport. The TE transport coefficients, such as electrical conductivity *σ*, Seebeck coefficient S, and thermal conductivity *κ*, for electrons or holes are determined by their energy spectra and chemical potentials. For instance, *σ* and *κ* are even as a function of E − *μ* with E being the energy spectrum, while S is an odd function with respect to E − *μ*. Therefore, when *μ* changes from a positive value to a negative one, the signs of *σ* and *κ* are not changed while only the sign of S is changed^37^. In addition, these TE transport coefficients are also dependent on the carrier velocity v_*k*_ = 1/*ħ* · ∂*E*/∂*k* with k being the wave vector. Due to different k-dependence features of the electron and hole energy spectra, the electron and hole velocities have different values, which can also lead to a difference in the TE transport coefficients as the chemical potential is varied from lying in the conduction band to lying in the valence band.

**Figure 2:**
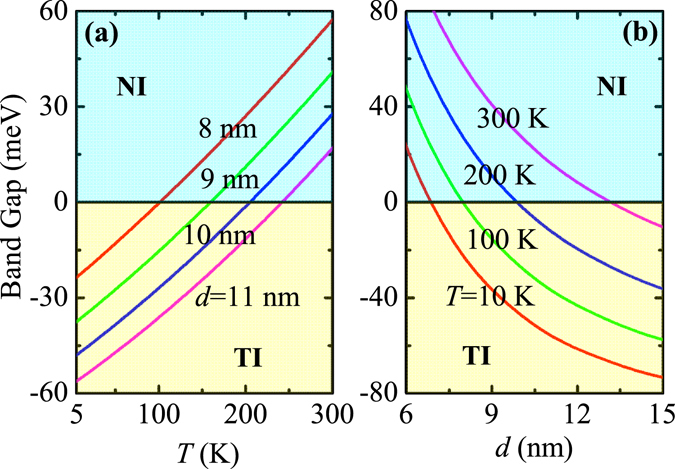
QW band gap as a function of thickness *d* (temperature *T*) for different temperatures *T* (thicknesses *d*) as indicated. Here, the light-cyan and light-yellow shadow regions denote the normal insulator (NI) phase and topological insulator (TI) phase, respectively.

In Fig. 2, we show the dependence of band gap of CdTe/HgTe QW on temperature (*T*) and thickness (*d*). As can be seen, by decreasing temperature and/or increasing thickness, the band gap changes continuously from the positive value to the negative one. This indicates a quantum phase transition between normal insulator (NI) and TI taking place in the QW system. Therefore, both temperature and thickness can effectively drive a TI phase in CdTe/HgTe QWs. At higher temperatures, the larger thickness is required to drive the QW system into the TI phase. These results are consistent with those published previously^42^.

**Figure 3:**
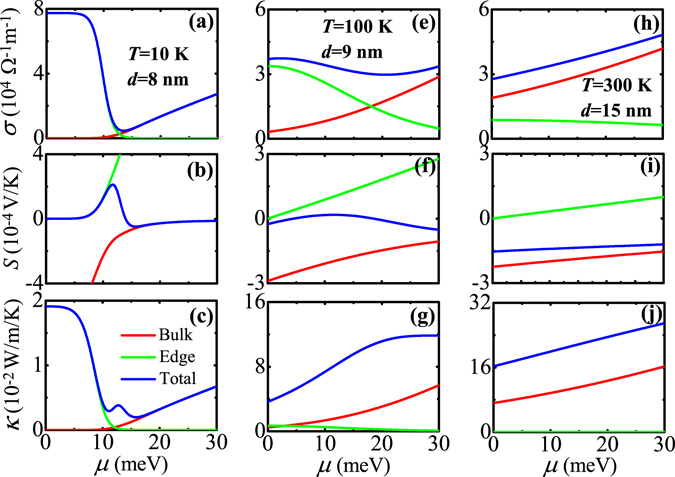
Electric conductivity, Seebeck coefficients, and electron thermal conductivity as a function of the chemical potential for various QWs in the TI regime at different temperatures, where (**a**)~(**c**) plot the results for *T* = 10 K and *d* = 8 nm, (**e**)~(**g**) for *T* = 100 K and *d* = 9 nm, and (**h**)~(**j**) for *T* = 300 K and *d* = 15 nm. Here, the black dashed line represents the bulk band-edge, the red and green solid lines represent the edge-state and bulk-state contributions to the TE transport, respectively, and the blue solid line represents the total TE transport.

To clearly demonstrate the temperature-driven TI phase in CdTe/HgTe QWs, a powerful tool is to calculate the energy spectrum of ribbon structure and to see whether there exists edge states or not. We take the ribbon width *w* = 400 nm and the QW thickness *d* = 8 nm. In Fig. 1(a) and (b), we show the effect of temperature on the energy spectrum of ribbon structure. It is clear that with deceasing temperature, a pair of gapless edge states occurs in the band gap formed by bulk states. In such a case, when the chemical potential of the system is tuned across the bulk band-edge, the edge and bulk states would exhibit quite distinct TE transport properties, as will be seen in the later. The characteristic features for edge and bulk states can be more clearly demonstrated by their probability density distributions |Ψ_edge_(*x*, *y*)|^2^ and |Ψ_bulk_(*x*, *y*)|^2^ in the plane of ribbon structure. We plot these results in Fig. 1(c) and (d). As can be seen, the edge and bulk states mainly distribute at the ribbon boundary and interior, respectively. The results shown in Fig. 3 well confirm that the temperature can effectively drive a TI phase in CdTe/HgTe QWs.

We now begin to discuss the TE transport properties of temperature-driven 2D TIs realized from CdTe/HgTe QWs. The TE transport coefficients such as the electrical conductivity *σ*, Seebeck coefficient *S*, and electron thermal conductivity *κ* are calculated as a function of the chemical potential *μ* for various QWs in the TI regime at different temperatures. The calculated results are shown in Fig. 3, where (a)~(c) plot the results for *T* = 10 K and *d* = 8 nm, (e)~(g) for *T* = 100 K and *d* = 9 nm, and (h)~(j) for *T* = 300 K and *d* = 15 nm. In all subfigures, the ribbon width of 2D TI is fixed at *w* = 500 nm. From Fig. 3, we can see the following interesting features. (1) Regardless of low or high temperatures, the contributions from edge and bulk states to the total *σ* are simply additive but to the total *S* and *κ* are not, which is well reflected by expressions of such three quantities [see Eqs (6a), (6b) and (6c)]. Edge and bulk states compete with each other to give the total TE transport properties. (2) In the low-temperature case (*T* = 10 K), due to gapless (gapped) property of edge (bulk) states, the edge states dominate the transport when *μ* is in the bulk band gap while the bulk states become dominant when *μ* is in the bulk band. As a result, when *μ* is tuned from lying inside the bulk band gap to entering into the bulk band, *σ* for edge (bulk) states decreases (increases) with increasing *μ*. The changing behaviors of *S* and *κ* for edge and bulk states as a function of *μ* could be understood with the help of Mott’s formula (*S*−*σ* relation) and Wiedemann-Franz law (*κ*−*σ* relation) in the adiabatic approximation, which read^43^

17a $$S=-{L}_{0}eT{[\frac{d\mathrm{ln}\sigma (E)}{dE}]}_{E=\mu },$$

17b $$\kappa ={L}_{0}T\sigma (\mu ),$$

where ${L}_{0}={\pi }^{2}{k}_{B}^{2}\mathrm{/(3}{e}^{2})$ is the Lorentz number. As mentioned before, *σ* for edge (bulk) states decreases (increases) with increasing *μ*. Thus, by analyzing *σ* as a function of *μ*, one can see that (i) *S* for edge (bulk) states is positive (negative) as a function of *μ*, (ii) its absolute value |*S*| for edge (bulk) states increases (decreases) with increasing *μ*, and (iii) *κ* for edge (bulk) states has the same variation trend as *σ* for edge (bulk) states. In particular, due to the competition between edge and bulk transports, the total *S* exhibits a peak structure when *μ* is near the bulk band-edge. (3) In the middle-temperature case (*T* = 100 K), the changing behaviors of *σ* and *κ* for edge and bulk states as a function of *μ* are the same as those in the low-temperature case. However, the Seebeck coefficients for edge and bulk states tend to cancel each other because they have opposite signs, leading to a very small value of the total *S*. (4) In the high-temperature case (*T* = 300 K), the edge-state transport is overwhelmed by the bulk-state transport. This is because more bulk states around the chemical potential take part in the TE transport at relatively high temperatures due to the small bulk band gap and large thermal broadening energy (characterized by *k*_*B*_ *T*). The total *σ*, *S*, and *κ* display the bulk-state behavior in the whole region of *μ*. This implies that the total TE transport properties at relatively high temperatures are governed by bulk states regardless of *μ* lying inside the bulk band gap or entering into the bulk-state region. (5) With increasing temperature, *σ* and *κ* for edge states decrease significantly. According to the expressions of transport integrals ${L}_{j}^{e}$ (*j* = 0, 1, 2) for edge states [see Eq. (12)], as temperature is increased, the integral intervals [−Δ_*b*_/(2*k*_*B*_ *T*), −Δ_*e*_/(2*k*_*B*_ *T*)] and [Δ_*e*_/(2*k*_*B*_ *T*),Δ_*b*_/(2*k*_*B*_ *T*)] in these expressions are reduced. Thus*, σ* and *κ* for edge states decrease with increasing temperature since they are proportional to transport integrals. Mathematically, the reduction of integral intervals by increasing temperature is equivalent to that by decreasing bulk band gap, by considering such an equation as Δ/(*k*_*B*_ T*) = Δ*/(*k*_*B*_ *T*) with T* and Δ* being effective temperature and bulk band gap, respectively. Therefore, as temperature is increased, the effective bulk band gap is decreased (let T* = *T*), and so the number of in-gap edge states (modes) is reduced, which is the physical reason why *σ* and *κ* for edge states decrease with increasing temperature.

**Figure 4:**
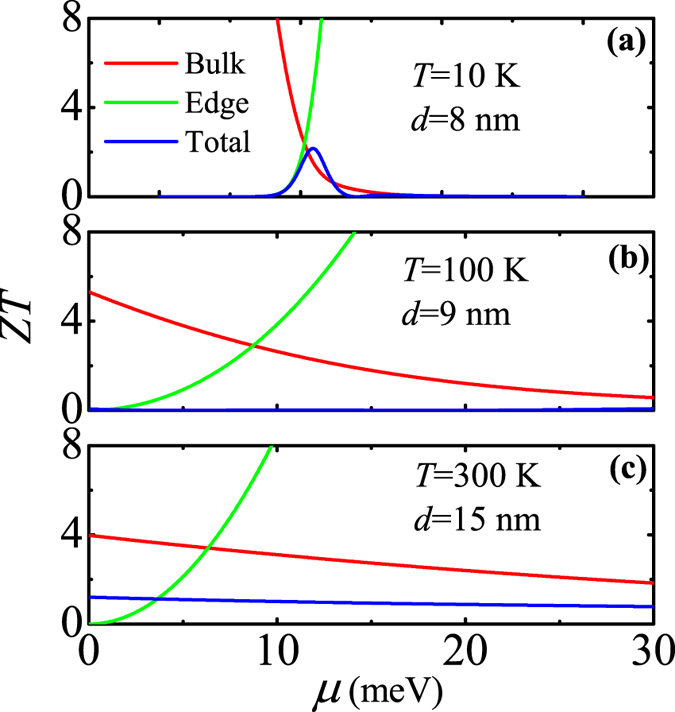
TE figure of merit *ZT* as a function of the chemical potential for various QWs in the TI regime at different temperatures: (**a**) *T* = 10 K and *d* = 8 nm, (**b**) *T* = 100 K and *d* = 9 nm, and (**c**) *T* = 300 K and *d* = 15 nm. Here, the black dashed line represents the bulk band-edge, the red and green solid lines represent the edge-state and bulk-state contributions to the TE transport, respectively, and the blue solid line represents the total TE transport.

Based on the above TE transport coefficients *σ*, *S*, and *κ*, the TE figure of merit *ZT* is calculated for the same QWs in the TI regime at different temperatures. The calculated results is shown in Fig. 4. In all subfigures (a)~(c), the ribbon width of the 2D TI is fixed at *w* = 500 nm. As can be seen, (1) in the low-temperature case (*T* = 10 K), due to the bulk-edge competition, *ZT* exhibits a peak structure when *μ* is near the bulk band-edge; (2) in the middle-temperature case (*T* = 100 K), regardless of *μ* in the bulk band gap or in the bulk band, the bulk and edge contributions to the TE transport tend to cancel each other, leading to an almost vanished *ZT*; and (3) in the high-temperature case (*T* = 300 K), due to the dominance of bulk states in the TE transport, *ZT* displays the bulk-state behavior in the whole region of *μ*. The interesting behaviors of *ZT* at different temperatures are well manifested by those of *S* at different temperatures (see Fig. 3). Here we give a reasonable explanation for such a manifestation: According to Eqs (6) and (7), the expression of *ZT* can be rewritten as *ZT* = *TσS* ^2^/*κ*. From this expression, one can see that *ZT* is mainly determined by *S* since (1) *ZT* is proportional to *S* ^2^ and (2) *κ*/(*σT*) is nearly a constant (approximated to be the Lorentz number) according to Wiedemann-Franz law.

**Figure 5:**
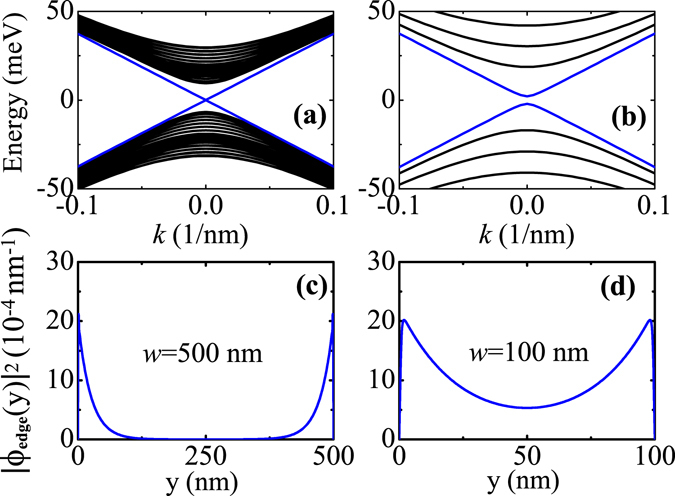
Energy spectra and edge-state wave functions at *k* = 0 for different ribbon widths: (**a**) and (**c**) plot the results for *w* = 400 nm; (**b**) and (**d**) plot the results for *w* = 100 nm. Here, the red dashed and solid lines in (**a**) and (**b**) represent the bulk band-edge and edge band-edge, respectively. The temperature and QW thickness are fixed at *T* = 100 K and *d* = 9 nm.

Next, we turn to discuss the dependence of TE transport properties on ribbon width of the temperature-driven 2D TI. To proceed, we first have a look at how the ribbon width influences the band structure of the 2D TI. In the calculation, we take the temperature *T* = 100 K and the QW thickness *d* = 9 nm. As demonstrated before, such a QW system is in the TI phase. In Fig. 5, we show the energy spectra for two different ribbon widths *w* = 500 and 100 nm. We can see that with decreasing *w*, the edge states at two boundaries of the 2D TI ribbon can hybridize to open a finite gap of several meV in the edge-state energy spectrum due to the substantial coupling of edge-state wave functions at such two boundaries. This is the so-called finite-size effect in the 2D TI^11^. The hybridization gap is smaller than the bulk band gap, as can be seen in the figure. When the chemical potential is tuned from lying inside the hybridization gap to entering into the bulk band, it sequentially pass through the edge band-edge and bulk band-edge. Moreover, with decreasing *w*, the bulk band gap is increased and the number of bulk bands is reduced due to the finite-size effect. These interesting features would have a great impact on the TE transport properties of temperature-driven 2D TIs, as will be clearly seen in the following.

**Figure 6:**
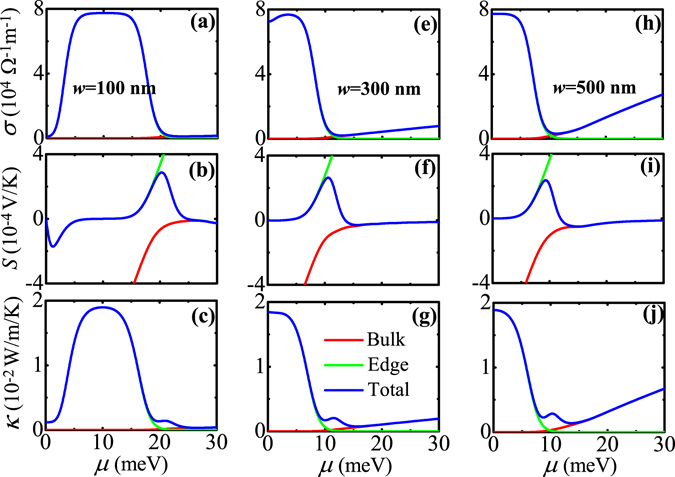
Electric conductivity, Seebeck coefficients, and electron thermal conductivity as a function of the chemical potential for various temperature-driven 2D TIs with different ribbon widths, where (**a**)~(**c**) plot the results for *w* = 100 nm, (**e**)~(**g**) for *w* = 300 nm, and (**h**)~(**j**) for *w* = 500 nm. Here, the black solid (dashed) line represents the edge (bulk) band-edge, the red and green solid lines represent the edge-state and bulk-state contributions to the TE transport, respectively, and the blue solid line represents the total TE transport. The temperature and QW thickness are fixed at *T* = 10 K and *d* = 7.5 nm.

In Fig. 6, we show the TE transport coefficients *σ*, *S*, and *κ* as a function of the chemical potential *μ* for various temperature-driven 2D TIs with different ribbon width *w*, where (a)~(c) plot the results for *w* = 100 nm, (e)~(g) for *w* = 300 nm, and (h)~(j) for *w* = 500 nm. In all subfigures, the temperature and QW thickness are fixed at *T* = 10 K and *d* = 7.5 nm, corresponding to the QW system in the TI regime. From Fig. 6, we can see the following interesting features. (1) When *μ* is near edge band-edge, *σ* and *κ* for edge states decrease with decreasing *w*. As is known, by decreasing *w*, the finite-size effect can be induced and thus the edge states can hybridize to open a finite gap in the edge-state energy spectrum. Due to the presence of such a hybridization gap, the transmission coefficient *T*(*E*) is reduced [see Eq. (9)] and as a result, *σ* and *κ* for edge states are decreased. (2) With decreasing *w*, the bulk band gap is increased and the number of bulk bands is reduced due to the finite-size effect. As a result, *σ* and *κ* for bulk states also decrease with decreasing *w*. (3) With decreasing *w*, the edge-state *S* changes the sign from minus to plus when *μ* is tuned from lying inside the bulk band gap to entering into the bulk band. Such a changing behavior for the edge-state *S* as a function of *μ* can be understood by analyzing the edge-state *σ* as a function of *μ* with the aid of Mott’s formula.

**Figure 7:**
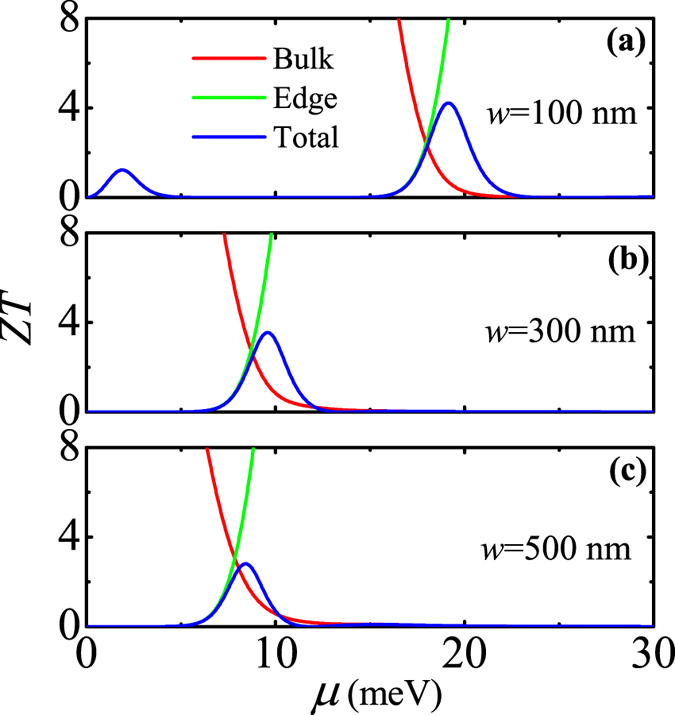
Low-temperature *ZT* as a function of the chemical potential for various 2D TIs with different ribbon widths: (**a**) *w* = 100 nm, (**b**) *w* = 300 nm, and (**c**) *w* = 500 nm. Here, the black solid (dashed) line represents the edge (bulk) band-edge, the red and green solid lines represent the edge-state and bulk-state contributions to the TE transport, respectively, and the blue solid line represents the total TE transport. The temperature and QW thickness are fixed at *T* = 10 K and *d* = 7.5 nm.

In Fig. 7, we show the dependence of low-temperature *ZT* on ribbon width *w* of the 2D TI. The temperature and QW thickness are taken as *T* = 10 K and *d* = 7.5 nm. As mentioned before, when the chemical potential *μ* is around the bulk band-edge, the low-temperature *ZT* exhibits a peak structure due to the bulk-edge competition. With decreasing *w*, the finite-size effect plays a significant role in the low-temperature *ZT*. Such an effect can give rise to the edge-state hybridization and so-produced hybridization gap. Therefore, one can see from this figure that for the narrowest ribbon width (*w* = 100 nm), the low-temperature *ZT* exhibits two peaks. Among them, one occurs around the bulk band-edge due to the bulk-edge competition around the bulk band-edge and the other occurs around the edge band-edge due to the edge-state hybridization. As *w* is increased, one of two peaks disappears due to the vanished edge-state hybridization gap and the other shifts to the lower chemical potential due to the reduced bulk band gap.

**Figure 8:**
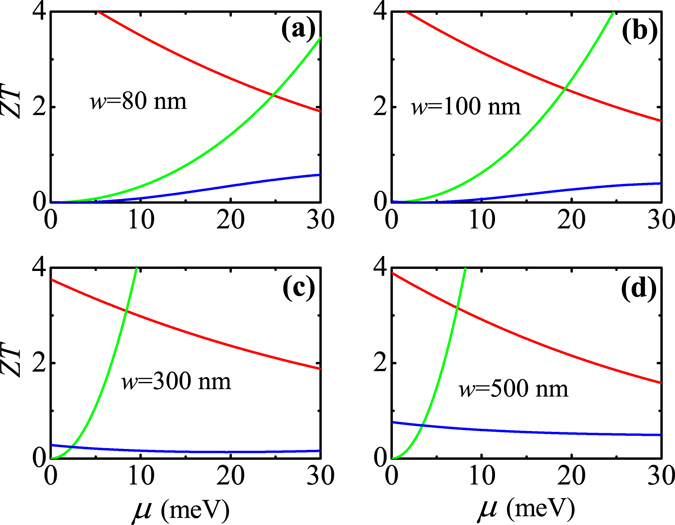
High-temperature *ZT* as a function of the chemical potential for various 2D TIs with different ribbon widths: (**a**) *w* = 80 nm, (**b**) *w* = 100 nm, (**c**) *w* = 300 nm, and (**d**) *w* = 500. Here, the black solid (dashed) line represents the edge (bulk) band-edge, the red and green solid lines represent the edge-state and bulk-state contributions to the TE transport, respectively, and the blue solid line represents the total TE transport. The temperature and QW thickness are fixed at *T* = 250 K and *d* = 13 nm.

In Fig. 8, we show the dependence of high-temperature *ZT* on ribbon width *w* of the 2D TI. The temperature and QW thickness are taken as *T* = 250 K and *d* = 13 nm. As can be seen, the *w*-dependence of high-temperature *ZT* has a totally different behavior as compared to that of low-temperature *ZT* shown in Fig. 7. With decreasing *w*, the high-temperature *ZT* first exhibits the bulk-state behavior and then the edge-state behavior, indicative of a bulk-to-edge transition in the TE transport. This is because the number of bulk states is proportional to the ribbon width due to the finite-size effect and thus the edge states in the narrower ribbon can have comparable or even larger contribution compared to bulk states.

## Concluding Remarks

In this work, we have theoretically investigated on the TE transport properties of temperature-driven 2DTIs realized from CdTe/HgTe QWs. We have started from a realistic band structure model and then calculated the TE transport coefficients on the basis of band structure results. It has been found that the temperature has two main effects that have a great impact on the TE transport properties of edge and bulk states in the 2D TI. The first and the most important one is that it can effectively drive a quantum phase transition between NI and TI. The second one is that it can significantly change the edge-carrier and bulk-carrier populations in the 2D TI. Through analyzing the TE transport coefficients such as the electric conductivity, Seebeck coefficient and thermal conductivity, we have found that the edge states dominate the TE transport at relatively low temperatures while the bulk states become dominant at relatively high temperatures. The TE figure of merit *ZT* has a peak structure around the bulk band-edge in the low-temperature case due to the competition between edge and bulk transports and this peak vanishes in the high-temperature case due to the dominance of bulk states in the TE transport. Moreover, we have also examined the dependence of TE transport properties of edge and bulk states on ribbon width of the 2D TI. Interestingly, we have observed that with decreasing ribbon width, the low-temperature *ZT* exhibits two peaks, among which one occurs due to the bulk-edge competition and the other occurs due to the edge-edge hybridization induced by finite-size effect; while the high-temperature *ZT* first exhibits the bulk-state behavior and then the edge-state one, indicative of a bulk-to-edge transition in the TE transport. Our theoretical results have demonstrated that in order to obtain the high *ZT* in designing thermoelectric devices, one should consider the following two points. (i) The smaller for 2DTI ribbon widths, the higher for *ZT* at low temperature, because that can suppress the phonon conductions. (ii) The nonmagnetic impurity density must be high to achieve the edge-states dominating the TE transport, comparing with the bulk states. The theoretical results may lead to a basic understanding of thermoelectric properties of 2D TIs. These systematic results could be relevant for potential applications of 2D TIs in high-performance thermoelectric devices.
